# Myocardial infarction with nonobstructive coronary arteries associated with blood transfusion in a young woman: A case report

**DOI:** 10.1097/MD.0000000000044328

**Published:** 2025-09-05

**Authors:** Hongshuo Chu, Ting Gao, Yang Li, Peng Zhong, Hongli Zhang, Chuanming Feng, Zixiu Wei

**Affiliations:** a Department of Cardiology, Jining Key Laboratory of Metabolic Cardiovascular Diseases, Institute of Cardiovascular Diseases of Jining Medical Research Academy, Jining No. 1 People’s Hospital, Jining, Shandong, China; b Department of Rehabilitation Medicine, Jining No. 1 People’s Hospital, Jining, Shandong, China.

**Keywords:** anemia, blood transfusion, case report, coronary angiography, myocardial infarction with nonobstructive coronary arteries

## Abstract

**Rationale:**

Myocardial infarction with nonobstructive coronary arteries (MINOCA) has diverse ischemic etiologies and has been defined by the absence of angiographically significant obstructive coronary artery disease. Blood transfusion has seldom been reported as a precipitating factor for MINOCA. Here, we present a rare case of transfusion-associated MINOCA in a young woman without underlying chronic conditions, aiming to raise clinical awareness of this uncommon yet important phenomenon and to explore its potential pathophysiological mechanisms.

**Patient concerns:**

A 30-year-old woman with acute anemia secondary to abnormal uterine hemorrhage developed MINOCA and cardiogenic shock following blood transfusion.

**Diagnoses:**

Abnormal uterine hemorrhage, anemia, MINOCA, cardiogenic shock.

**Interventions:**

The patient received hemocoagulase and oxytocin to control vaginal bleeding. Anemia was managed with a total transfusion of 6 units of erythrocytes. Coronary angiography was performed promptly after the diagnosis of acute coronary syndrome. Vasopressin was administered to manage cardiogenic shock. Clopidogrel, ticagrelor, nicorandil, and rosuvastatin were prescribed to promote the recovery of coronary microvascular function.

**Outcomes:**

Electrocardiographic changes resolved within hours, and left ventricular ejection fraction improved from 44% to 66% by 1 month. The patient completed a 6-minute walk test without symptoms and remained stable, with resolution of anemia and full cardiac recovery.

**Lessons:**

The delicate balance between hemorrhage and hemostasis was emphasized. Treatment strategies for blood transfusion-induced MINOCA remain limited, and our therapeutic approach may provide an effective option for clinical practice.

## 1. Introduction

Anemia is a critical contributor to the imbalance between myocardial oxygen supply and demand, potentially leading to type 2 myocardial infarction (MI).^[1]^ Blood transfusion is an effective intervention to correct anemia-induced metabolic imbalance. MI with nonobstructive coronary arteries (MINOCA) is defined as MI without angiographic stenosis >50%.^[2]^ Type 2 MI and MINOCA often present with similar clinical features. However, blood transfusion may induce MINOCA, although such cases are rarely reported. In this study, we present a rare case of blood transfusion-induced MINOCA and describe the therapeutic approach undertaken.

## 2. Case description

A 30-year-old woman was admitted to the hospital with vaginal bleeding, exhaustion, and dyspnea. The patient had been diagnosed with ovarian endometriosis and had undergone surgery, followed by a two-month course of ethinylestradiol and cyproterone acetate prior to admission. No other surgical history was apparent, except for a cesarean delivery 6 years earlier. The patient had no history of chronic diseases or cardiovascular risk factors. Smoking and drug abuse were denied. Initial vital signs were normal except for low blood pressure (86/60 mm Hg). Blood clots were observed in the vaginal canal. The initial parameters were reported as follows: hemoglobin = 68 g/L (reference range 120–130 g/L), hematocrit = 18.8% (reference range 35–45%), mean corpuscular volume = 91.7 fL (reference range 82–100 fL), erythrocyte count = 2.05 × 10^12^/L (reference range 3.8–5.1 × 10^12^/L), white blood cell count = 7.54 × 10^9^/L (reference range 3.5–9.5 × 10⁹/L), platelet count = 231 × 10^9^/L (reference range 150–450 × 10⁹/L). Electrocardiography demonstrated no significant abnormalities.

**Figure 1.**
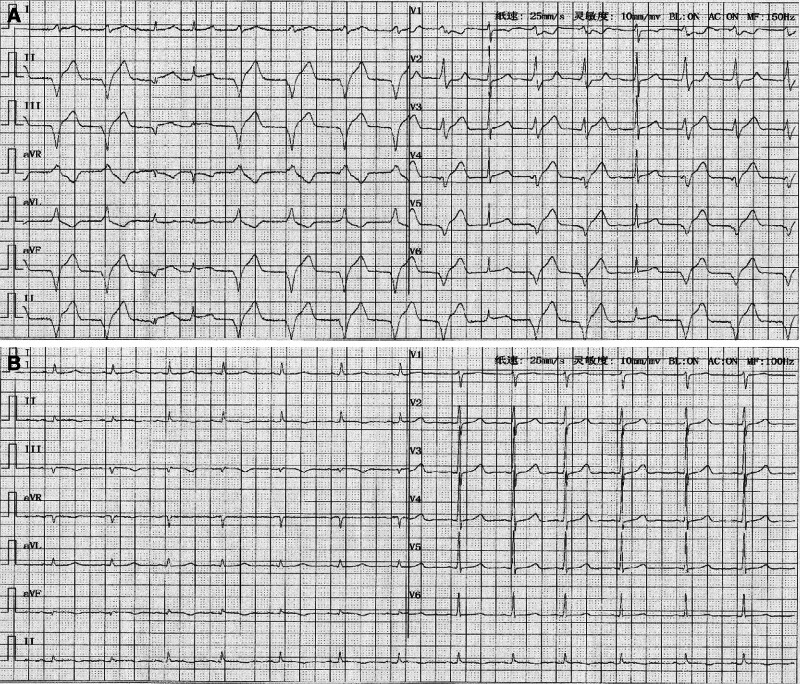
The evolution of electrocardiographic changes. (A) At the occurrence of chest pain, ECG demonstrated accelerated ventricular tachycardia and ST-segment elevation in leads II, III, and aVF. (B) ECG was normal 9 hours after chest pain. ECG = electrocardiogram.

**Figure 2.**
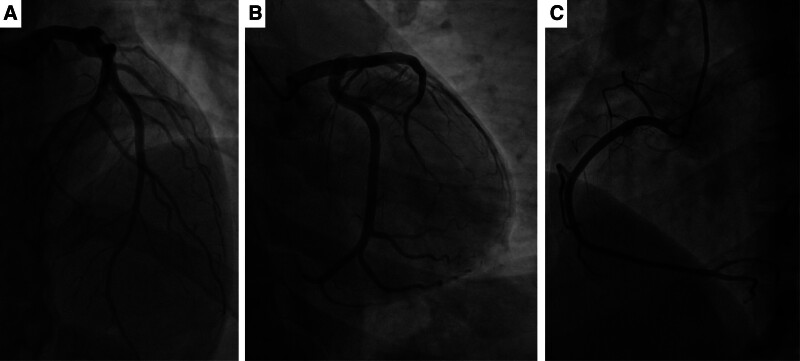
Coronary angiography showing no abnormality of the left (A, B) and right (C) coronary arteries.

The etiology of moderate anemia was attributed to abnormal uterine hemorrhage, as determined by a gynecologist. Hemocoagulase and oxytocin were used to control the bleeding, while the daily doses of ethinylestradiol and cyproterone acetate tablets were maintained. Following the transfusion of 4 units (1000 mL) of crossmatched type O Rh-positive erythrocyte suspension over 4 hours, the patient reported relief of fatigue and dyspnea. Twenty-four hours later, an additional 2 units of erythrocyte suspension were transfused without immediate adverse events. However, 4 hours after the second transfusion, the patient developed severe retrosternal chest pain and dyspnea. The patient’s blood pressure remained low (88/53 mm Hg), and irregular heart rhythms were noted on auscultation. Electrocardiography revealed accelerated ventricular arrhythmia with ST-segment elevation in leads II, III, and aVF (Fig. 1A). Laboratory investigations showed an elevated N-terminal prohormone brain natriuretic peptide concentration of 308 pg/mL (reference range: 0–125 pg/mL) and a cardiac troponin I concentration of 1.21 ng/mL (reference range: 0–0.019 ng/mL). Acute coronary syndrome was diagnosed. After administration of loading doses of aspirin (300 mg) and clopidogrel (300 mg), emergent coronary angiography was performed (Fig. 2). Notably, there was no evidence of obstruction, plaque formation, vasospasm, dissection, or slow flow in either the left or right coronary arteries. Due to the patient’s hemodynamic instability, optical coherence tomography (OCT) was not performed in order to minimize procedural time. Consequently, we assumed that microvascular dysfunction was the major cause of the subsequent diagnosis of MINOCA. Soon after, the patient developed signs of cardiogenic shock, including cold, clammy skin and worsening dyspnea. Management included antiplatelet therapy with aspirin and clopidogrel, statin therapy with rosuvastatin, vasodilation therapy with nicorandil, and hemodynamic support with vasopressin. Electrocardiography performed 9 hours after symptom onset demonstrated resolution of ST-segment elevation and the absence of arrhythmias (Fig. 1B). However, transthoracic echocardiography revealed hypokinesis of the inferior and posterior walls of the left ventricle, along with a reduced left ventricular ejection fraction of 44%.

After ten days of treatment, the patient’s cardiac function fully recovered, and vasopressin was discontinued. Prior to discharge, the patient successfully completed a 6-minute walk test, covering more than 600 m without symptoms. Upon discharge, the patient was prescribed clopidogrel, nicorandil, and rosuvastatin. At 1-month follow-up, transthoracic echocardiography revealed normal left ventricular wall motility and an ejection fraction of 66%. Additionally, anemia had nearly resolved, with a hemoglobin concentration of 118 g/L.

**Figure 3.**
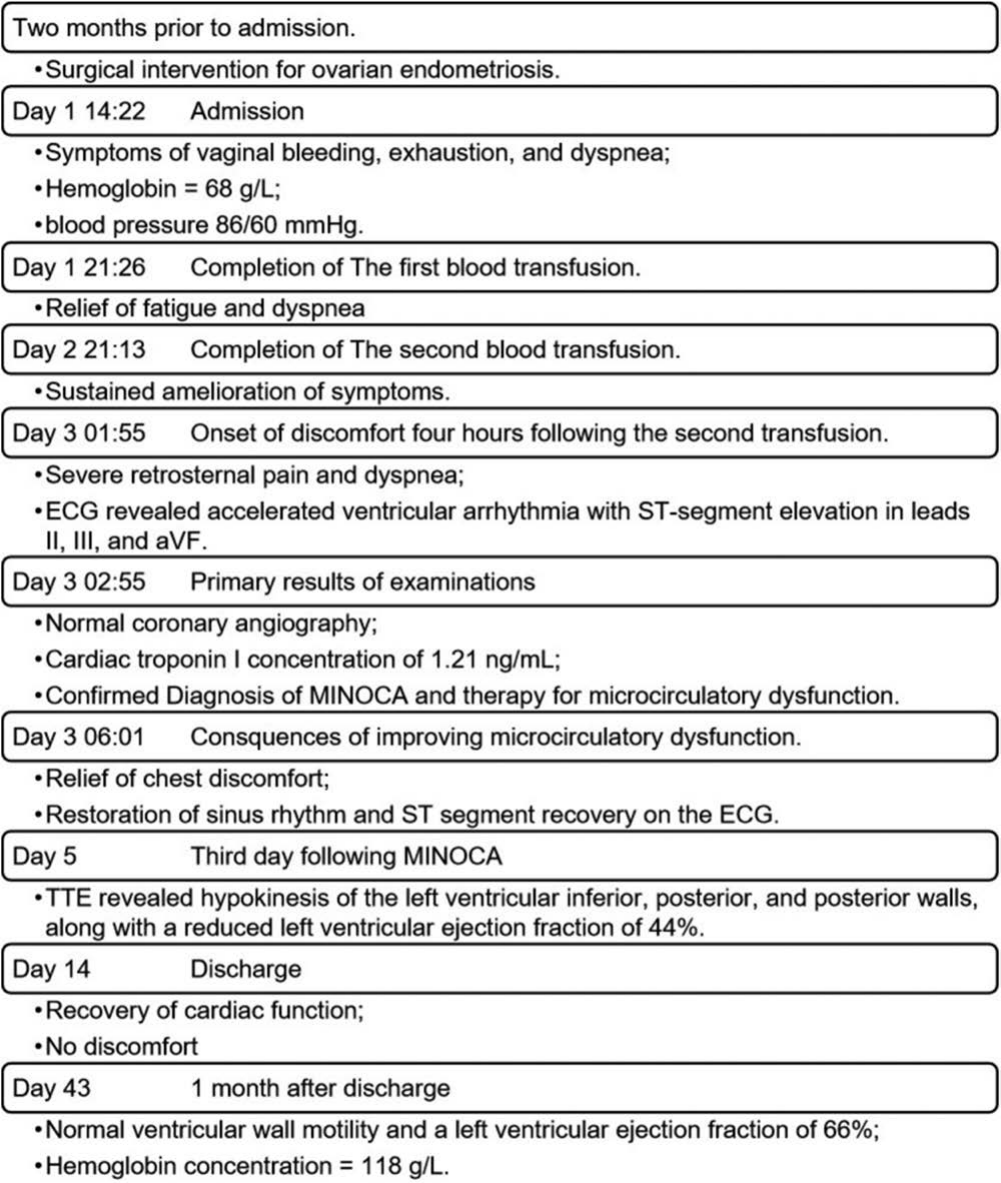
Historical and current information from this episode of care organized as a timeline. ECG = electrocardiogram, MINOCA = myocardial infarction with nonobstructive coronary arteries, TTE = transthoracic echocardiography.

This paper presents the development of events using a timeline (Fig. 3) to enhance the reader’s understanding of their chronological sequence and interrelationships. This study was approved by the Ethics Committee of Jining No. 1 People’s Hospital. Written informed consent was obtained from the patient for publication of this case report and any accompanying images.

## 3. Discussion

The prevalence of MINOCA is much lower than that of MI with obstructive coronary arteries on angiography (≥50% stenosis of a major epicardial vessel).^[1,2]^ According to the fourth definition of MI, MINOCA can be classified into 3 types. Type 1 is associated with thromboembolic events caused by plaque rupture or erosion and calcified nodules. Type 2 occurs as a result of an imbalance between oxygen demand and supply by coronary factors (such as coronary spasm, microvascular dysfunction, spontaneous coronary dissection, or micro-thrombosis) and/or noncoronary factors (such as arrhythmia, hypertension, shock, or anoxia). Type 3 occurs with sudden cardiac death and absence of ≥50% stenosis of an epicardial artery on autopsy.^[3]^ OCT can aid in the identification of underlying coronary pathology, while cardiac magnetic resonance imaging provides further characterization of myocardial injury.^[4,5]^

Anemia is one of the etiological factors for type 2 MI.^[3]^ However, the patient in the present study developed MI after receiving erythrocyte suspension transfusion. Therefore, MINOCA was considered the working diagnosis instead of type 2 MI, due to the resolution of anemia following transfusion.^[6]^ Nonetheless, MINOCA associated with blood transfusion has rarely been reported. Velibey et al described a case of a 62-year-old male with chronic anemia secondary to short gut syndrome who developed MINOCA following routine erythrocyte suspension transfusion. The patient was successfully treated with dual antiplatelet therapy, metoprolol, and nitrates, achieving a favorable recovery.^[7]^ Another study reported transient ST-segment elevations occurring twice after transfusion of concentrated erythrocytes prior to surgery.^[8]^ In a young woman with hypertension, MINOCA secondary to type 2 spontaneous coronary artery dissection was diagnosed through coronary angiography, OCT and cardiac magnetic resonance imaging.^[9]^ Notably, in all 3 aforementioned cases, chronic comorbidities appeared to have contributed to the development of MINOCA.

There is a paucity of studies reporting the development of MINOCA associated with blood transfusion in young women without underlying chronic disease. In this study, we observed no evidence of coronary spasm, dissection, plaques, or thrombosis in the epicardial arteries on coronary angiography. Moreover, there were no signs of coronary slow flow induced by higher microcirculatory resistance, which helped to exclude the possibility of microvascular thrombosis. Cardiogenic shock, decreased left ventricular ejection fraction, hypokinesis on transthoracic echocardiography, and accelerated ventricular tachycardia all suggested severe cardiac complications. Therefore, the diagnosis of MINOCA was confirmed based on ST-segment elevation observed on electrocardiography.

There are several limitations to our study. First, cardiac magnetic resonance imaging, provocative spasm testing, and OCT, which could have provided additional valuable insights, were not performed due to rational clinical considerations.^[2,5]^ Second, the lack of long-term follow-up limits our ability to evaluate the sustained prognosis and recurrence risk. Third, the mechanism of transfusion-induced MINOCA cannot be confirmed based solely on observational data and requires further investigation to elucidate the association.

Vasopressin was used to stabilize the patient’s hemodynamics, and we effectively treated the patient with a regimen that included nicorandil, rosuvastatin, and dual antiplatelet therapy. The resolution of ST-segment elevation indicated improved microcirculatory function. After reviewing the therapeutic regimen, safe doses of drugs, such as hemocoagulase and oxytocin, were unlikely to contribute to the development of MINOCA. Type 2 MINOCA was therefore thought to be caused by blood transfusion-induced microvascular spasm, and nicorandil helped to alleviate microcirculatory dysfunction, as described previously.^[2,10,11]^ Patients with MINOCA are increasingly recognized by clinicians and generally exhibit favorable prognoses.^[6,12]^ Consistently, our patient responded well to treatment and gradually achieved complete recovery.

Of note, antithrombotic therapy may increase the risk of hemorrhage in patients with MI, while ischemia may be worsened by hemostasis. Antiplatelet therapy may prevent the patient from coronary microvascular thrombosis. Therefore, careful consideration must be given to balancing these therapeutic strategies. Fortunately, in this case, we successfully controlled the patient’s vaginal bleeding before the onset of MINOCA.

## 4. Conclusions

Blood transfusion is commonly recommended for the treatment of type 2 MI induced by anemia; however, it rarely precipitates MINOCA. Coronary angiography and the provocative spasm test are advocated to differentiate the underlying pathogenesis, although the risks associated with these examinations should be carefully assessed in advance. The mechanisms underlying blood transfusion–induced MINOCA remain poorly understood; nevertheless, effective treatment for microcirculatory dysfunction may help to improve prognosis.

## Author contributions

**Conceptualization:** Hongshuo Chu, Zixiu Wei.

**Data curation:** Hongshuo Chu, Ting Gao, Yang Li, Hongli Zhang, Chuanming Feng.

**Investigation:** Hongshuo Chu, Ting Gao, Peng Zhong, Hongli Zhang.

**Methodology:** Hongshuo Chu, Zixiu Wei.

**Project administration:** Hongshuo Chu.

**Supervision:** Zixiu Wei.

**Validation:** Hongshuo Chu, Ting Gao.

**Writing – original draft:** Hongshuo Chu, Ting Gao.

**Writing – review & editing:** Hongshuo Chu, Ting Gao, Zixiu Wei.
